# How to engineer aerosol particle properties and biopharmaceutical performance of propellant inhalers

**DOI:** 10.1016/j.ijpharm.2023.122676

**Published:** 2023-03-05

**Authors:** Precious Akhuemokhan, Natalie Armstrong Green, Allen Haddrell, David Lewis, Jonathan P. Reid, Ben Forbes

**Affiliations:** a Institute of Pharmaceutical Science, King’s College London, London SE1 9NH, UK; b School of Chemistry, University of Bristol, Bristol BS8 1TS, UK; c Oz-UK Limited, Corsham, Wiltshire SN13 9BY, UK

**Keywords:** Biopharmaceutics, Dissolution, Humidity, Maturation, Beclomethasone, PreciseInhale, Electrodynamic balance

## Abstract

Given the environmental compulsion to reformulate pressurised metered dose inhalers (pMDI) using new propellants with lower global warming potential, this study investigated how non-volatile excipients can be used to engineer aerosol particle microphysics and drug release. The dynamics of change in particle size, wetting and physical state were measured for single particles (glycerol/ethanol/beclomethasone dipropionate; BDP) in the aerosol phase at different relative humidity (RH) using an electrodynamic balance. BDP dissolution rates were compared for aerosols from pMDI containing different ratios of BDP:glycerol or BDP:isopropyl myristate (IPM). In 45 % RH, ethanol loss was followed by evaporation of condensed water to generate spherical particles with solid inclusions or compact irregular-shaped solid particles, according to the presence or absence of glycerol. In RH > 95 %, condensed water did not evaporate and BDP formed solid inclusions in water/glycerol or water droplets. Varying the non-volatile component, 0–50 % w/w, in pMDI resulted in a concentration-dependent 4–8-fold reduction in BDP dissolution rate. These findings demonstrate that non-volatile excipients provide a means of engineering aerosol properties and, modifying the rate of drug release from aerosol medicines. We also demonstrated differences between particles formed *in vitro* in ambient humidity versus higher humidity, more like that encountered during oral inhalation.

## Graphical abstract

**Figure:**
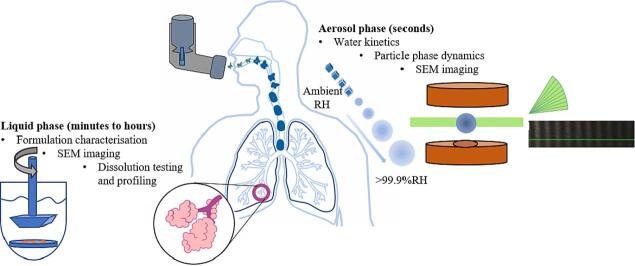

## 1 Introduction

Aerosol medicines are widely used to treat respiratory disease and pressurised metered dose inhalers (pMDI) are the most economic and commonly used inhalation delivery devices. Although it is only 20 years since ozone-depleting chlorofluorocarbons were replaced by hydrofluoroalkanes (HFA), reformulation of current pressurised metered dose inhalers to use new propellants with lower global warming potential may be required as soon as 2025 to meet clinical, economic and climate needs (Pritchard, 2020). This is a significant challenge and, simultaneously an opportunity for product innovation; for example, by extending the use of formulation approaches to engineering aerosol properties and inhaled product performance.

Typical solution pMDI formulation mixtures include the active pharmaceutical ingredient, a cosolvent (commonly ethanol) and a volatile propellant (Lewis, 2007, Myrdal et al., 2014). When the current hydrofluoroalkane (HFA) propellants were introduced, non-volatile excipients (NvE) such as glycerol were incorporated to modulate the aerodynamic particle size distribution of aerosols produced by some solution inhalers to match the aerosol properties of the original chlorofluorocarbon products (Ganderton et al., 2002, Woodcock et al., 2002). Glycerol modifies the particle microphysics of aerosols from solution pMDIs by increasing particle size, modifying the morphology and solid state of BDP aerosols (Buttini et al., 2014). Particle maturation during aerosol generation and inhalation occurs on a time scale of a few seconds, which corresponds to clinical advice to patients, to inhale slowly and deeply through their pMDI and breath hold for up to 10 s or as long as they are comfortable (American Journal of Respiratory and Critical Care Medicine, 2014)

The electrodynamic balance (EDB) is a single particle measuring technique that can collect extensive data on the maturation kinetics of drug/excipient droplets in the aerosol phase and provide unique insights into the size, wetting and phase state of inhaled particles over a relevant timescale (Legh-Land et al., 2021, Haddrell et al., 2019, Haddrell et al., 2014). The volatility of propellants makes direct measurements of propellant droplets in the EDB impractical, e.g. HFA134a evaporates within 0.004 s for droplets < 10 µm in size (Stein and Myrdal, 2006). However, an ethanol cosolvent evaporates considerably more slowly than the propellant, e.g. 0.2 s (Gregson et al., 2019) and thus can be used reliably to generate single droplets for studying the aerosol microphysics as particles mature over the timescale of 0.1–10 s. Furthermore, the relative humidity in the particle trap can be adjusted to explore the interaction of aerosol with water vapour and the water transport kinetics experienced by the particle during generation and inhalation (Haddrell et al., 2017, Myrdal and Angersbach, 2008).

Although a key determinant in inhaled drug bioavailability is regional aerosol deposition in the lungs which is aerodynamic particle size dependent, dissolution in the lung lining fluid and uptake into lung epithelial tissue determine the duration and extent of exposure (Forbes et al., 2015). Unlike drug dissolution/precipitation within the aerosol phase, which occurs in seconds, bulk phase dissolution, which dictates drug release into the body after deposition onto the lung wall, occurs on the scale of minutes to hours depending on the solubility of the compound (Siepmann and Siepmann, 2013). *In vitro/ex vivo* research into pMDI solution formulations of beclomethasone dipropionate (BDP), a drug for asthma treatment, has suggested non-volatile excipients in these formulations may affect drug disposition after aerosol deposition in the lungs. Freiwald et al. (2005), compared two HFA formulated BDP pMDI products, Qvar® and Sanasthmax®, which both contained ethanol but differed in the presence of glycerol. Using a simple dialysis model, a slower release of drug from lung tissue to perfusate by the glycerol-containing formulation was observed. Grainger et al. (2012) investigated the *in vitro* dissolution and absorptive transfer across epithelial cell layers for the Qvar and Sanasthmax inhalers, although multiple variables included aerodynamic and geometric particle size distributions, solid state and inclusion of glycerol. A follow-on study in which two test formulations were created to exhibit identical aerosol properties apart from the presence of glycerol (Lewis et al., 2014), also found differences in dissolution profiles and drug permeability across cell layers (Haghi et al., 2014), pointing to an effect of glycerol, the non-volatile excipient (NvE) on drug kinetics beyond size-dependent particle deposition and dissolution.

The aim of the study was to use the EDB to investigate how glycerol and humidity influence particle conformation during maturation and explore the impact of two NvE on the rate of drug release from pMDI aerosol particles. Specific objectives were to (i) simulate BDP aerosol particle formation from droplets containing ethanol ± glycerol using single particle measurements to study excipient effects on size and phase state dynamics, (ii) evaluate the impact of relative humidity on BDP particle development and wetting, (iii) formulate pMDI with systematically varying glycerol and isopropyl myristate (IPM) concentrations to determine the effect of each excipient on aerosol particle dissolution. Isopropyl myristate has been suggested by a patent (Meakin et al., 2008) to modulate aerosol particle size in a similar manner to glycerol. Thus, it was included in this study to act as a comparator to glycerol. With these techniques, the complete timeline of the inhaled drug, from particle generation through maturation to drug release and pharmacokinetics, can be explored.

## 2 Methods

### 2.1 Single aerosol particle dynamics

**Fig. 1:**
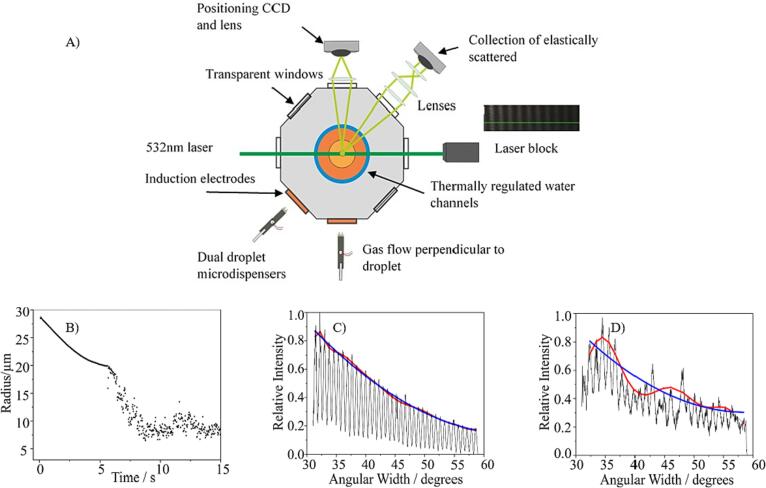
Panel (A) is a schematic representation of the electrodynamic balance used to measure the crystallisation and dissolution dynamics of particles formed from the BDP solutions. Panels (B), (C) and (D) illustrate examples of data interpretation from phase functions. In (B), the radius of an evaporating droplet can be estimated over time from the phase function of each time point. The physical state of the droplet can be determined by comparing the quality of fits of the polynomial (red line) and quadratic (blue line) curves to the peak intensity maxima. In (C), a particle is identified as homogeneous when the peaks are uniform and both fits are good and in (D), a particle is identified as a spherical liquid droplet containing inclusions when the peak spacings are regular but the intensities are irregular, and the quadratic fit is poor. (For interpretation of the references to colour in this figure legend, the reader is referred to the web version of this article.)

Test solutions were formulated to contain BDP/ethanol ± glycerol and were named according to the mass percentage of the non-volatile component that was glycerol. To illustrate, the glycerol-free formulation was composed of 97.5 % ethanol and 2.5 % BDP—‘glycerol 0 %’, whilst the glycerol-containing formulation contained 95 % ethanol with 2.5 % BDP and 2.5 % glycerol—‘glycerol 50 %’. A comparative-kinetics electrodynamic balance (EDB) was used to measure the dynamics of droplets formed from each solution at different relative humidities (RH) at 20 °C (Davies et al., 2012). The EDB has been described in detail by Rovelli et al. (2016). Briefly, a micro-dispenser was used to generate droplets of initial radius of 25–30 µm. Droplets were charged upon generation by an induction electrode held at constant DC bias placed in front of the dispenser tip. Droplets were captured in the central chamber of the EDB (Fig. 1) within an AC electric field. The temperature was maintained at 20 °C by recirculating a mixture of water and ethylene glycol (50 % v/v) from a thermostatic water bath and through the top and bottom of the chamber (Rovelli et al., 2016). The RH was adjusted by mixing humidified and dry nitrogen flows to achieve conditions from 0 % to 95 % RH. Measurements for each droplet type and RH were made for 10–20 droplets.

Once in the trap, the droplet was illuminated with light from a green laser (532 nm), which was elastically scattered. The near-forward scattered light is collected over a range of angles, centred at 45° (Rovelli et al., 2016) and recorded as a series of interference fringes, referred to as the phase function. The angular spacing of the fringes is used to estimate droplet size using the geometric ray optics approximation (Kulmala et al., 1992). Sizing accuracy was ± 100 nm when the droplet was homogenous and spherical. Particle radius and phase were analysed using custom-written phase analysis software. Although accurate sizing was not possible once a particle departed from a homogeneous structure, the form of the phase functions could be used to identify a particle as either a homogenous spherical droplet, a droplet containing inclusions or a solid/non-spherical solid particle (Haddrell et al., 2019).

### 2.2 Morphology of particles analysed by EDB

A specialised trap, *controlled electrodynamic levitation and extraction of bioaerosol onto a substrate*, CELEBS (Fernandez et al., 2019), was used to capture particles for SEM imaging. CELEBS enables populations of droplets (∼30) to be produced using the same temperature and RH conditions as those used in the EDB. BDP droplet evaporation profiles collected on the EDB indicated that crystallisation occured within 1.5 min. To allow sufficient time for crystallisation, droplets were thus held in CELEBS for 2 min and then deposited on a statically charged petri dish before imaging. Particle samples were collected for two drying conditions, broadly defined as rapid crystallisation (drying at 0 % RH) and slow crystallisation (drying at 85 %RH) to match the evaporation profiles measured in the EDB studies at 20 °C. This was carried out for glycerol-free (glycerol 0 %) and glycerol-containing (glycerol 50 %) BDP solutions.

### 2.3 Propellant inhaler formulations and aerosol characterisation

The proportional relationship between the MMAD (µm) of an HFA134a solution aerosol population and the non-volatile component, *n* (%w/w), has been empirically derived (Lewis, 2007):

(1) $${\text{MMAD}}_{134}\approx 2.31\;n^{1/3}$$

In subsequent work (Lewis et al., 2014), formulations were designed to vary in the content of non-volatile excipient but produce similar aerodynamic size distribution by modifying the propellant, the actuator orifice geometry and metering volume (Brambilla et al., 1999). In this present study, a series of five formulations for glycerol and isopropyl myristate (IPM) containing varying ratios between non-volatile excipient and drug (Table 1), was designed using similar principles to produce formulations with similar aerosol particle size distribution and fine particle fraction (proportion of aerosol particle population below 5 µm in size).

**Table 1:** Composition of glycerol and isopropyl myristate test formulations.

BDP content % w/w	NvE (Glycerol/Isopropyl myristate) content % w/w	Ratio between NvE content and BDP content	NvE as a percentage of non-volatile component %
0.35	0	0:100	0
0.35	0.04	10:90	10
0.35	0.09	20:80	20
0.35	0.15	30:70	30
0.35	0.23	40:60	40
0.35	0.35	50:50	50

To prepare each formulation, BDP powder (Pharmaceutical secondary standard grade, from Sigma-Aldrich) was first dissolved in ethanol. Glycerol or isopropyl myristate was then added to the bulk solution. The drug solutions containing NvE were transferred to 19 mL Presspart® Aluminium C128 canisters which were then fitted with Bespak® BK 357 metering valves. After crimping, the canisters were filled with the HFA134a propellant to the required mass. The filled canisters were stored at room temperature for the duration of testing. For characterisation, each canister was placed into a 630 series Bespak actuator and fired into an Andersen Cascade Impactor (*ACI*) fitted with a USP metal throat induction port for aerodynamic characterisation using a flow rate of 28.3 L/min. BDP was quantified by UPLC/MS using a Waters Acquity UPLC with QDA system (Waters Ltd, Elstree, UK). After quantification, the data was analysed with the CITDAS V3.10 software (Copley UK). The metered dose, delivered dose, fine particle dose, fine particle fraction and MMAD were determined from the cascade impactor data.

### 2.4 Dissolution of aerosols from propellant inhalers

The inhalers were actuated into the 300 mL holding chamber of the PreciseInhale® (Inhalation Sciences, Stockholm Sweden) via a USP induction Port No 1 at ambient humidity and temperature, typically 32 % RH and 22 °C. A 9-stage Marple cascade impactor, with the intermediate impactor stages removed, was attached to the outlet. Using a flow rate of 61.8 L/min, BDP particles were deposited at the last stage on a glass cover slip (for imaging) or a glass microfibre filter grade GF/F (for dissolution testing). To evaluate particle morphology, the particles collected were imaged using a scanning electron microscope (Hitachi High-Technologies Corporation, Japan). For dissolution testing, the particle-containing filters were attached to a watch-glass and transferred to a USP 2 dissolution apparatus containing surfactant (SDS 0.25 % w/v) in a phosphate buffer solution at 37 °C with paddle speed of 50 rpm. 1 mL samples were withdrawn over the course of an hour and replaced with an equivalent volume of fresh dissolution medium. BDP content was quantified by HPLC. Dissolution profile modelling was carried out using the Weibull function as described by Franek et al. (2018)

(2) $$F_{d}=1-e^{-{\left(t/a\right)}^{b}}$$

Where *F_d_* is the cumulative fraction of drug dissolved at time *t*, *a* is replaced with the time factor *T_d_* indicating the time taken for 63.2 % of BDP to dissolve and *b* is the dissolution profile shape factor (Langenbucher, 1972).

### 2.5 Quantification of BDP by reverse-phase HPLC-UV

Quantification of BDP in dissolution samples was achieved using an RP-HPLC-UV system (waters 2795 Separations Module). Integration was carried out using an Empower Pro data analysis software. Separation was achieved using a Luna® C18(2) LC column (100 Å, 3 μm, 150 × 4.6 mm, Phenomenex, Cheshire, UK) which was maintained at 40 °C using a column heater. The mobile phase comprised of acetonitrile and HPLC-grade water in a ratio of 65:35 (v/v). The flow rate of the mobile phase was set at 1 mL/min. UV detection of BDP was at 254 nm, where the detection wavelength was determined using Waters 2296 Photodiode Array Detector following injection of a standard solution of BDP.

### 2.6 Data analysis

Data from the aerodynamic characterisation of inhalers for both excipients were analysed statistically using the 2-way ANOVA (GraphPad Prism 9.4) to test for significance where p-value < 0.05.

## 3 Results

### 3.1 Evaporation kinetics of glycerol and ethanol droplets

**Fig. 2:**
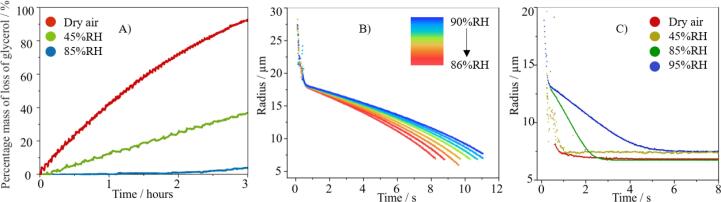
Evaporation profiles of individual single excipient and binary droplets trapped using an electrodynamic balance. (A) Percentage loss of mass of glycerol from a droplet versus time when held at 0 %, 40 % and 85 % RH, (B) Droplet radius versus time when a droplet of ethanol is suspended at 86 % (red) – 90 % (blue) RH. (C) Droplet radius versus time for a binary droplet (glycerol/ethanol 10/90  %w/w) trapped at 0 %RH, 45 %RH, 85 %RH and 95 %RH. (For interpretation of the references to colour in this figure legend, the reader is referred to the web version of this article.)

Aerosol phase measurements were made to establish the drying kinetics of the excipients, both individually and in combination excipients. Glycerol evaporation was studied at different RHs, ranging from dry air to 85 % RH. Although glycerol is non-volatile in the context of pharmaceutical inhaler use, evaporation does occur from small droplets of pure glycerol over time periods which may affect laboratory analyses if studies are not performed on fresh samples. At 20 °C, glycerol loss over 3 h as a proportion of mass was 93 % under dry conditions (0 %RH), 37 % in ambient conditions (45 % RH) and 4 % in humid conditions (85 % RH) (Fig. 2a). The different rates of mass loss at different RHs reflect the dependence of equilibrium vapour pressure on composition through Raoult’s law: a decrease in the proportion of glycerol in a droplet at elevated RH leads to a reduction in component mole fraction, vapour pressure and thus, mass flux.

Ethanol cosolvent evaporation was measured at 86–90 % RH (Fig. 2b). Previous studies have shown that a pure ethanol droplet transforms into a pure water droplet in < 0.5 s, subsequently evaporating at the rate expected for pure water (Gregson et al., 2019). The initial radius of each droplet was ∼ 25 µm, which reduced to ∼ 17.5 µm in under 1 s, corresponding to ethanol transferring into the gas phase with evaporative cooling leading to the condensation of water and the formation of a water droplet. Thereafter, water evaporated from the droplet at a rate inversely related to RH, reflecting the varying vapour phase concentration gradient between the droplet surface and gas phase driving the diffusional transport of water away from the droplet.

Droplets of glycerol/ethanol (10/90 % w/w) were trapped at RHs of 0, 45 85 and 95 % relative humidity. In dry air, a spherical, homogeneous droplet of glycerol is rapidly formed. At 45–95 % RH, the droplet remained homogenous and spherical with water accretion driven by the evaporative cooling from ethanol evaporation producing a miscible water/glycerol droplet. The evaporation kinetics were consistent with the rapid evaporation of ethanol followed by evaporation of condensed water at a rate inversely related to the RH. The change in evaporation rate, at a radius of ∼ 13 µm, was relatively small at 45 % RH compared to glycerol evaporation in dry air, but increasingly marked at RH of 85 % and 95 % (Fig. 2c).

### 3.2 Maturation of BDP/ethanol ± glycerol droplets at varying RH

**Fig. 3:**
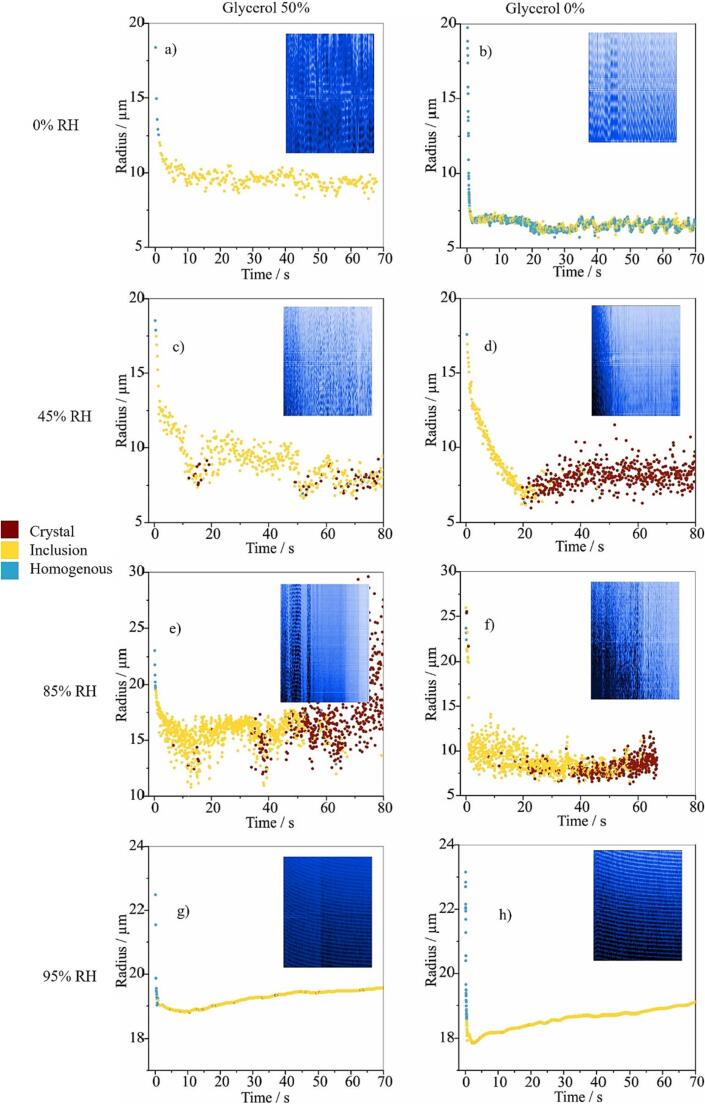
The evaporation of ethanol and condensation of water on droplets with compositions of glycerol 50 % (left column) and glycerol 0 % (right column) at varying RH: dry air (0 %RH, A & B), ambient conditions (45 %RH, C & D), humid conditions (85 %, E & F) and wet conditions (95 %, G & H). The phase of the particle is identified at every time point: homogenous (blue), inclusions (yellow) and solid (dark red). The inset figures show qualitatively the evolving light scattering phase function over the same timescale with time on the abscissa and scattering angle on the ordinate axis. (For interpretation of the references to colour in this figure legend, the reader is referred to the web version of this article.)

Evaporation profiles over 80 s for representative individual droplets are presented with size measurements and phase functions to indicate particle morphology and points of phase transition (Fig. 3). The reproducibility of the droplet measurements was demonstrated in a collation of all data from a large number of individual droplet measurements (Supplementary Information, SI 1).

The initial rapid decrease in radius was complete within 0.2 s from droplet generation and occurred at the same rate independent of relative humidity and starting composition. This was attributed to the evaporation of ethanol and causes concomitant condensation of water, when present, from the surrounding vapour phase via evaporative cooling (Gregson et al., 2019). In dry air, the formulation containing BDP and glycerol was observed to form a droplet containing solid inclusions at a radius ∼ 1/2 of the initial droplet size (Fig. 3a), suggesting that solid BDP inclusions are suspended within a spherical glycerol droplet, i.e. the angular light scattering profile retained uniformly distributed diffraction peaks but acquired significant noise in light scattering intensity (Haddrell et al., 2019). In comparison, the glycerol-free formulation produced a smaller compact particle composed entirely of BDP (Fig. 3b).

At RH 45 %, representing typical air humidity in a laboratory setting, the glycerol-containing formulation formed a droplet with inclusions within 20 s whereas glycerol-free formulation formed a solid particle within ∼ 18 s, once the water that condensed onto the droplet when ethanol was lost had evaporated (Fig. 3c and 3d). The particles with glycerol persisted as liquid droplets with solid inclusions throughout the measurement period. Although the size measurements reported have large uncertainty (±1.5 µm), a consequence of the inhomogeneity in particle phase and composition presenting challenges in interpreting the phase function, the data clearly show the timescale of the drying kinetics and the resulting phase state.

At 85 % RH, particles showed similar behaviour to those at 45 % RH but with noticeable differences attributable to the higher humidity. Both formulations produced initial signatures indicating spherical liquid droplets containing solid BDP inclusions; notably, the particles were larger, and the water loss phase was slower at 85 % RH compared to 45 %, resulting in a longer time for the particle to become predominantly solid (Fig. 3e and 3f). At 95 % RH, droplets from both formulations remained spherical in shape with solid inclusions of BDP (Fig. 3g and 3 h). The light scattering phase functions were considerably less noisy than those at lower RH, suggesting an almost homogeneous particle despite the inclusions. The angularly-resolved fringes can be tracked and host droplet sizes estimated. The presence of glycerol led to a larger equilibrated droplet size due to the hygroscopic growth after initial condensation (Fig. 3g; Cai et al., 2015). This process was confirmed by similar measurements of glycerol 20 % w/w droplets in ethanol at high RH (SI 2). The droplet formed from the glycerol-free solutions also remained spherical with the presence of inclusions and showed hygroscopic growth, albeit to a lesser degree. To retain and attract water, even at such high RH, some of the BDP must be at least partly solubilised within the aqueous phase, consistent with previous reports suggesting a hygroscopic growth factor of 1.2 at ∼ 95 % RH for BDP particles (Farkas et al., 2017). The growth of the equilibrated particle size was < 1 μm over 80 s, and reflects relatively slow mass transport of water in the vicinity of saturation RH, possibly influenced by an upward drift in humidity when using extremely elevated RH and slow dissolution of BDP inclusions into the aqueous droplet.

### 3.3 Morphology of BDP/glycerol particles

**Fig. 4:**
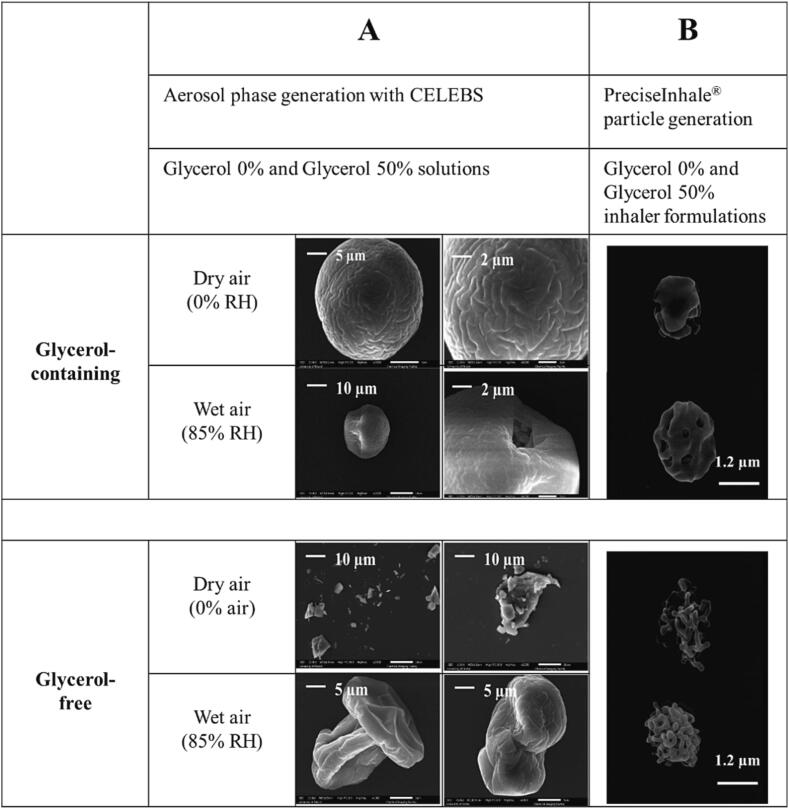
SEM imaging of BDP particles with and without glycerol. Comparisons are described between (A) BDP particles generated from solutions using CELEBS at two different relative humidity conditions and (B) BDP particles arising from test inhaler formulations created for the bulk dissolution studies.

Particles matured from the BDP solutions (±glycerol) used for single particle measurements were collected using CELEBS, under dry and moist (0 and 85 % RH) conditions and analysed by SEM to support the size and morphology information inferred from the electrodynamic balance measurements. By providing structural integrity during ethanol evaporation, the presence of glycerol leads to the formation of a spherical particle (Fig. 4a) consistent with BDP concentrating at the surface of a droplet and forming a shell. In the absence of glycerol, the BDP forms compact irregular solid particles as ethanol evaporates rapidly. In humid conditions, the water that partitions into the glycerol phase during particle formation is lost on exposure to the high vacuum in the SEM analysis, leading to buckling and deformation. Without glycerol, the particles formed are smaller, show considerable deformation and crumpling during SEM analysis, and consistent hollow BDP shell particles that deflate as water evaporates during secondary drying.

Particles generated from similar formulations in HFA134a propellant collected from pMDI actuated under ambient laboratory conditions (intermediate RH) as described in Section 2.4, are similar in appearance (Fig. 4b) to particles reported by others (Grainger et al., 2012, Lewis et al., 2014, Buttini et al., 2014). At ambient humidity, the glycerol-free particles appear more like those generated in dry air than at 85 % RH.

### 3.4 Propellant inhaler aerosol characteristics

**Fig. 5:**
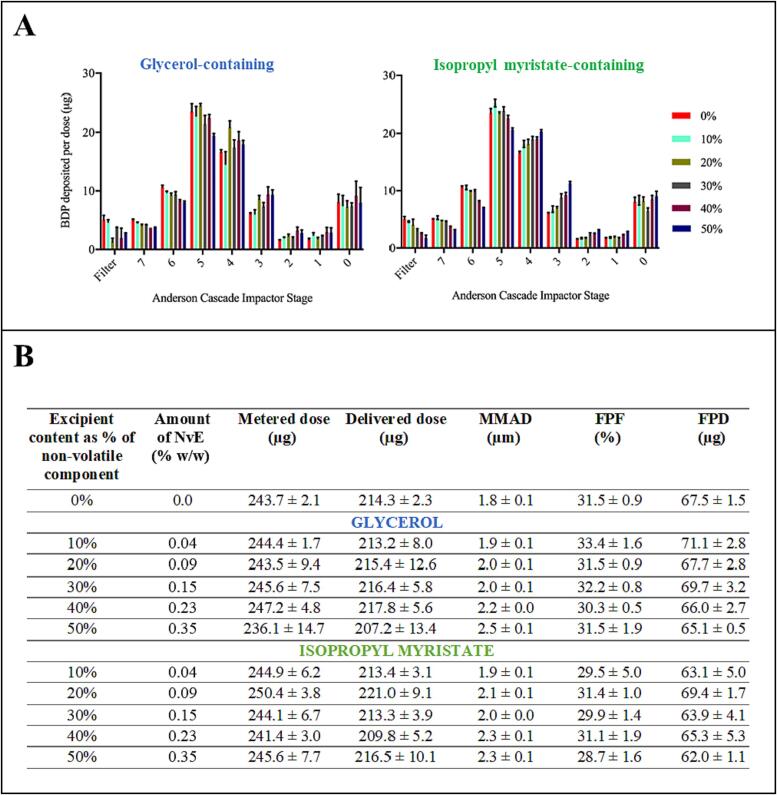
(A) Aerosol particle size distribution profiles for test pMDI formulations containing glycerol and isopropyl myristate as obtained from cascade impaction, and (B) Key aerosol population parameters for glycerol and isopropyl myristate test pMDI formulations. Data shown represents mean ± SD from n = 3 Andersen cascade impactor measurements.

**Fig. 6:**
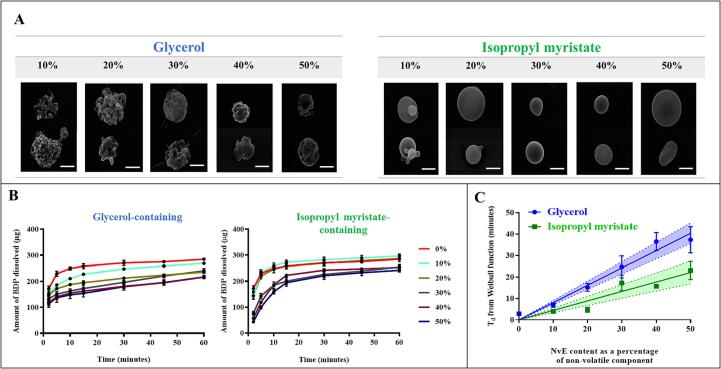
Particle morphology and dissolution behaviour. (A) Scanning Electron Microscopy images of particles collected from test formulations by the PreciseInhale® with varying % non-volatile excipient (NvE), glycerol or isopropyl myristate. Images were taken at × 25000 magnification. Scale bar = 1.2 µm. (B) Dissolution profiles of test formulations containing glycerol and isopropyl myristate after transfer to a USP 2 rotating paddle apparatus. Data represents mean ± SD (n = 3). (C) Weibull function T_d_ (the time interval taken for 63 % of drug to dissolve) change with increasing NvE content (n = 3). Line of best fit with 95 % confidence interval by GraphPad Prism USA 9.4.

HFA 134a pMDI formulations containing varying amounts of glycerol or IPM were designed to deliver 250 µg of BDP with each actuation. The aerodynamic particle size distribution of all formulations was equivalent (p > 0.05), although there was a trend towards increasing mass of BDP deposited in earlier stages of the *ACI* with increasing concentration of NvE (Fig. 5a). The aerosol properties were typical of orally inhaled drug products and were correspondingly similar across parameters including delivered dose, MMAD, fine particle fraction and fine particle dose (p > 0.05; Fig. 5b). SEM showed all particles were spherical, but differences were observed in surface texture. The surface topology of the glycerol-containing particles became smoother as the proportion of glycerol in the formulation increased, whereas the surface of IPM-containing particles was smooth at all concentrations (Fig. 6a).

### 3.5 Dissolution of BDP aerosols from propellant inhalers

The presence of glycerol or IPM reduced the dissolution rate of BDP (Fig. 6b). To compare the effects of concentration and excipient type, the Weibull function was employed to model the dissolution profiles and the time scale parameter T_d,_ was derived to describe the time taken for 63 % of drug to be released from the formulation (Franek et al., 2018, Langenbucher, 1972) (Fig. 6c). It was observed that the IPM-containing formulations consistently exhibited a faster rate of dissolution when directly compared with the glycerol formulation with the equivalent amount of excipient. The formulation without any NvE exhibited the lowest T_d_ of all the formulations tested. An inverse relationship between the amount of non-volatile excipient present in the formulation and the dissolution rate is observed for both glycerol and isopropyl myristate. The dissolution rate of the aerosol from the excipient-free formulation exceeded that of all the formulations containing glycerol and was similar to the IPM formulations that had the lowest amounts of excipient, namely IPM 10 % and IPM 20 %.

## 4 Discussion

BDP, glycerol, and ethanol formulations were selected for this study because they are clinically relevant and build on previous work exploring the role of glycerol in BDP-containing solution pMDI. Upon actuation of a pMDI, the expansion of propellant drives particle formation in a dynamic process in which there is rapid and dramatic composition and temperature change, making it difficult to study the speed of events and the environment in which this occurs. In this study, we used an electrodynamic balance to measure changes in size and the physical state of a single particle continuously at controlled humidity. This enabled the effects of glycerol on particle formation at different humidity to be investigated. We expanded the study by exploring systematically the effect of varying glycerol and IPM concentration on aerosol particle dissolution to provide an indication of the PK-modifying potential of non-volatile excipients.

The electrodynamic balance enabled the rapid evolution in particle size, composition (including wetting) and phase state of individual droplets that represent aerosols from pMDI, to be measured at varying RH. Initially, aerosol phase evaporation was measured for individual and binary solvent mixtures and showed that the ethanol cosolvent evaporates completely in < 1 s driving water condensation from the air due to evaporative cooling, followed by the evaporation of this condensed water at a rate determined by the surrounding RH and glycerol content. In a pMDI, the evaporative cooling effect would be compounded by the even quicker evaporation of the propellant (Legh-Land et al., 2021). For the BDP solutions, an initial reduction in size due to evaporation of ethanol was observed which was countered by condensation of water across all conditions except dry air, regardless of the composition of the formulation. Although glycerol itself is lost by evaporation, the timescale makes this insignificant during inhaled delivery although it should be considered if particles are being collected and processed for analysis. As drying data was not obtained for isopropyl myristate, it is unclear whether this excipient also evaporates over longer timeframes.

In dry air, glycerol-free BDP particles were angular in shape and present in a solid state as observed by SEM. In the presence of glycerol, the particles were spherical with solid BDP inclusions dispersed in a matrix. BDP did not dissolve fully in aerosol droplets formed in the presence of moist air despite the acquisition of water as volatile solvents evaporate. BDP was dispersed as insoluble inclusions within a water or water/glycerol droplet that lost water at a rate governed by the surrounding RH. Some pMDI formulations exhibited an irregular appearance possibly resulting from internal cavitation due to the rapid evaporation of volatile components when forming the aerosol particles (Zhu et al., 2014). Increasingly spherical particles were formed as the amount of glycerol increased from 10 to 50 % (Fig. 6), whereas IPM formulations produced spherical particles at all concentrations.

The high RH in the airways means that the particles will contain water through to the point of deposition. At 95 % RH (Fig. 3g & 3h), the phase functions indicated an inclusion droplet in which it is likely that BDP is partially dissolved as the phase functions indicate a more uniformed and spherical droplet than at 85 % RH or lower. Below 95 % RH, the formation of solid states within the particles is more apparent, although this happens more slowly in the presence of glycerol, which may hinder diffusion by conferring a higher internal viscosity within a droplet and slow molecular reorganisation. Similarly, previous studies have suggested that more viscous particles exhibit delayed responses to the moisture content in the gas phase as the water kinetics within the particle may be hindered (Haddrell et al., 2017, Bones et al., 2012).

As beclomethasone dipropionate has low aqueous solubility, lung exposure and absorption from the lungs may be anticipated to be dissolution rate dependent. Thus, under general frameworks for ‘clinically-relevant dissolution’ (McAllister et al., 2022) and specific proposals for an inhaled bioclassification system (Hastedt et al., 2022, Bäckman et al., 2022) dissolution may be considered a key quality attribute linked to clinical performance. Following evaporation of propellant and then ethanol, particles consist principally of the BDP and glycerol or isopropyl myristate, with a degree of water content as discussed above. The influence of particle chemistry and morphology on dissolution rate is well established (Mosharraf and Nyström, 1995), and having demonstrated the impact of glycerol on particle formation, we hypothesised that the amount and type of excipient would impact on the dissolution of BDP particles.

The rate of *in vitro* dissolution in the bulk liquid phase decreased with increasing non-volatile excipient content for both glycerol and isopropyl myristate (Fig. 6b). The dissolution rate may be affected both by the microphysical processes occurring on the particle during formation and the presence of the excipient during the bulk dissolution measurements. Unsurprisingly, given the differences in particle morphology observed by SEM, differences in dissolution rate reduction were observed with isopropyl myristate compared to the glycerol formulation equivalents (Fig. 6c). Part of the explanation may be the difference in viscosity between glycerol (1412 cP; Segur and Oberstar, 1951) and isopropyl myristate (5 cP; Rowe, Sheskey, and Quinn, 2009). BDP particles may be more homogenously dispersed in the less internally viscous isopropyl myristate droplets due to the microphysics of particle formation, which may then enhance dissolution compared to the glycerol equivalent.

The similarity of effects found for IPM to glycerol provides some evidence that the effects studied are generalisable for non-volatile excipients, while the differences provide the potential to extend the currently limited range of excipients suitable for inhalation to provide additional formulation options. To date, isopropyl myristate has not been incorporated into licensed inhaled formulations, but it has been shown to exhibit similar particle size modulating properties as glycerol with an average MMAD of 2.3 µm for the isopropyl myristate 50 % formulations compared with the commercial glycerol-containing Clenil® Modulite product MMAD of 2.5 µm (Ganderton et al., 2002). There may be scope for incorporating the excipient into the pressurised metered dose inhalers in the same manner as glycerol (Lewis et al., 2005).

Although the effects of formulation composition and relative humidity on particle morphology and dissolution observed in this study may vary for active pharmaceutical ingredients (API) with a different dose or solubility, a number of considerations are raised:

1. When used by patients, pMDI may be actuated into the humid environment of the mouth and drawn immediately into the lungs, where humidity approaches 100 %, or actuated into a spacer. In the former scenario, BDP particles will form in a warm, humid environment and will contain water when depositing into the lung lining fluid. With a spacer, particles form in the air of ambient temperature and humidity and have longer to mature before being inhaled. The results of our study suggest that aerosols from propellant inhaler products may have distinct quality differences when generated in each of these scenarios,
2. Unlike powder inhalers, aerosol characterisation for pMDI is complicated by respirable particles not being formed until the product is actuated. Our data illustrate the potential for pMDI particles to possess different properties (morphology, composition, solid state properties) according to (i) the environment in which they form, and (ii) how the aerosol is collected and processed before analysis or performance testing. Different methods may be appropriate according to whether the aerosol is being analysed for quality control purposes or to characterise the properties of particles that deposit in the lungs, e.g. for physiologically based biopharmaceutics modelling,
3. In new product development, it is a challenge to use consistent aerosol and formulations test articles through non-clinical to clinical translation and during transition through clinical phases. Our findings reinforce the importance of aligning the methodology used for (i) aerosol capture and analysis of pMDI products during pharmaceutical development – as described above, (ii) aerosol presentation to bioassay and safety/efficacy models, and (iii) administration in the clinic. Systems such as the PreciseInhale used in our study have been designed to enable the same aerosol to be collected for analysis, delivered to *in vitro* test systems, delivered to animals for safety and efficacy studies e.g. for accurate dosimetry of inhaled formulation to animals (Selg et al., 2012) and particle collection in dissolution studies (Gerde et al., 2017).

To summarise, it is recognised that particles collected and characterised *in vitro* after emission from pMDI using standard pharmacopeial and quality control methods may not represent entirely faithfully the particles that form and deposit in the lungs of patients. We have utilised an electrodynamic balance to study the effect of volatile and non-volatile excipients on the dynamics of particle maturation in different humidity and shown how this impacts the composition of aerosol particles formed. Our data shows that aerosol droplets generated by pMDIs from the formulations containing glycerol form solid BDP inclusions within host glycerol droplets with a water content governed by the RH to which they are exposed. Further, we have explored the effect of non-volatile excipients on dissolution, finding effects that preliminary physiologically based biopharmaceutics modelling (data not shown) indicates would markedly affect PK if replicated *in vivo.*

## 5 Conclusion

This study applied novel experimental techniques to reveal the processes occurring during the evolution of an inhaled aerosol drug particle from a model solution inhaler formulation along the full timeline of its journey through composition and physical state changes in the gas phase after pMDI actuation (i.e. the inhalation process) through to drug release upon introduction to a liquid phase (i.e. post-deposition performance). Single particle measurements in aerosol phase provided insights into how glycerol, a non-volatile excipient affects aerosol particle compositional change, microstructure, and morphology under ambient conditions typical of those found in laboratories conducting pharmaceutical product testing compared to the humid conditions which exist in the lungs of patients using inhaled medicines. Dissolution profiling of formulations with varying amounts and type of excipient showed a relationship between the amount of non-volatile excipient and dissolution rate that also depended on the type of excipient used. Effects of glycerol, which is a component of a number of licensed inhaled products and has been studied previously, were compared with an alternative aerosol particle bulking agent, isopropyl myristate, which was shown to confer similar properties.

Taken as a whole, the findings in this study demonstrate how excipients can be used to influence the particle properties of medical aerosols produced by pMDI and highlights the importance of studying these effects in a way that avoids artefacts due to differences between laboratory environments and those that pertain clinically.

## CRediT authorship contribution statement

**Precious Akhuemokhan:** Methodology, Investigation, Formal analysis, Writing – original draft. **Natalie Armstrong Green:** Methodology, Investigation, Formal analysis, Writing – original draft. **Allen Haddrell:** Methodology, Visualization. **David Lewis:** Conceptualization, Supervision, Resources, Software. **Jonathan P. Reid:** Visualization, Supervision, Conceptualization, Funding acquisition, Writing – review & editing. **Ben Forbes:** Conceptualization, Supervision, Funding acquisition, Project administration, Resources, Writing – review & editing.

## Declaration of Competing Interest

The authors declare the following financial interests/personal relationships which may be considered as potential competing interests: Precious Akhuemokhan reports financial support and equipment, drugs, or supplies were provided by Chiesi Ltd.

## Data Availability

Data will be made available on request.

## Appendix A Supplementary material

Supplementary Data 1 EDB measurements on ethanol, glycerol and BDP solutions (with or without glycerol).
