# Supplementary material for: How to engineer aerosol particle properties and biopharmaceutical performance of propellant inhalers
Source: Int J Pharm. 2023 Mar 5;634:122676. doi: 10.1016/j.ijpharm.2023.122676 (PMC10685293; doi:10.1016/j.ijpharm.2023.122676)
Supplement: Supplementary Data 1 — EDB measurements on ethanol, glycerol and BDP solutions (with or without glycerol). [file mmc1.pdf]

Supplementary information

EDB measurements on ethanol, glycerol and BDP solutions (with or without glycerol)

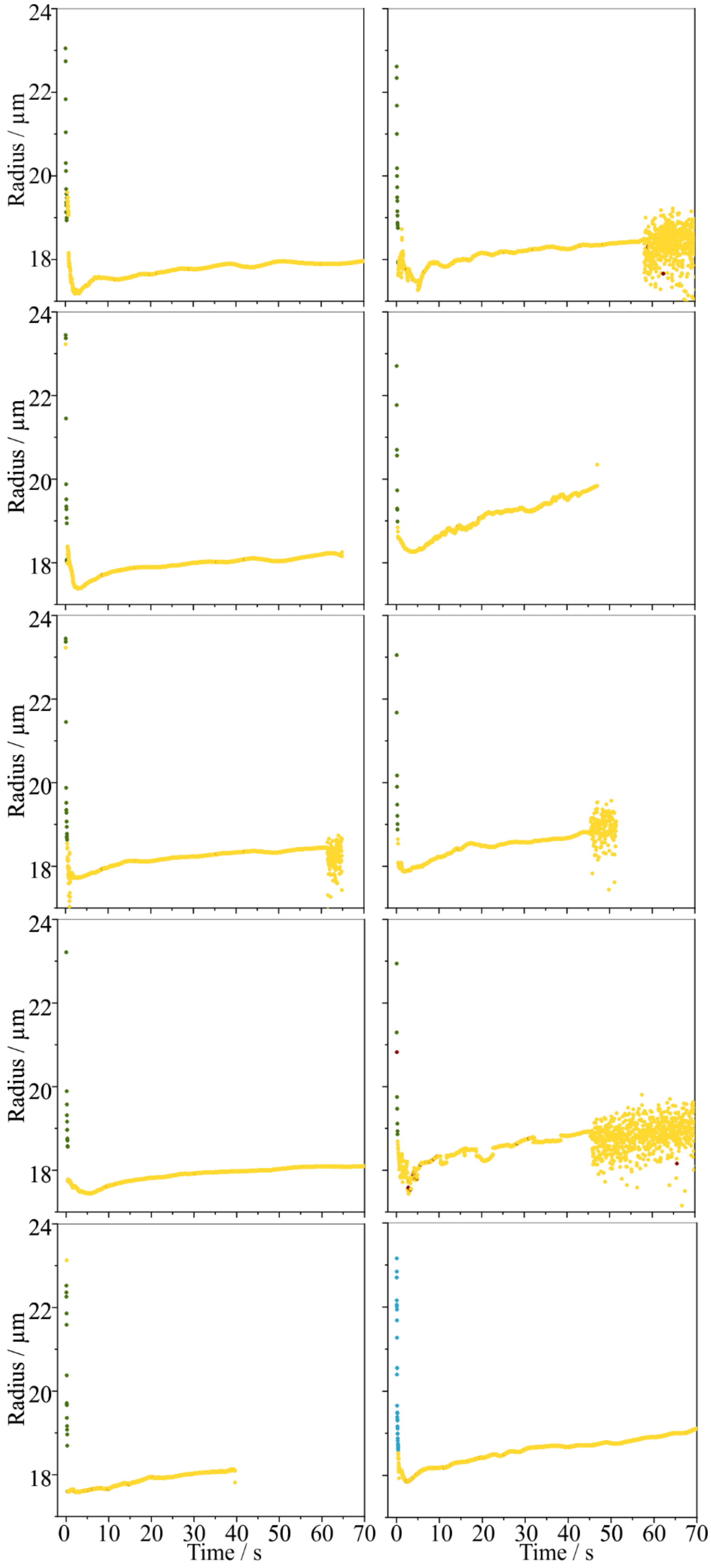

Figure S1. Droplet profiles of glycerol0% formulation at 95%RH, collected on an EDB. An inclusion droplet was formed in every case with a similar final size. Peak tracking has been used to correct for radius size. Note, noisy data points were not able to be corrected due to considerably more erratic light scattering. Bottom right figure is presented in the main text, Figure 3, as a fair representation of the phase dynamics for this formulation and conditions.

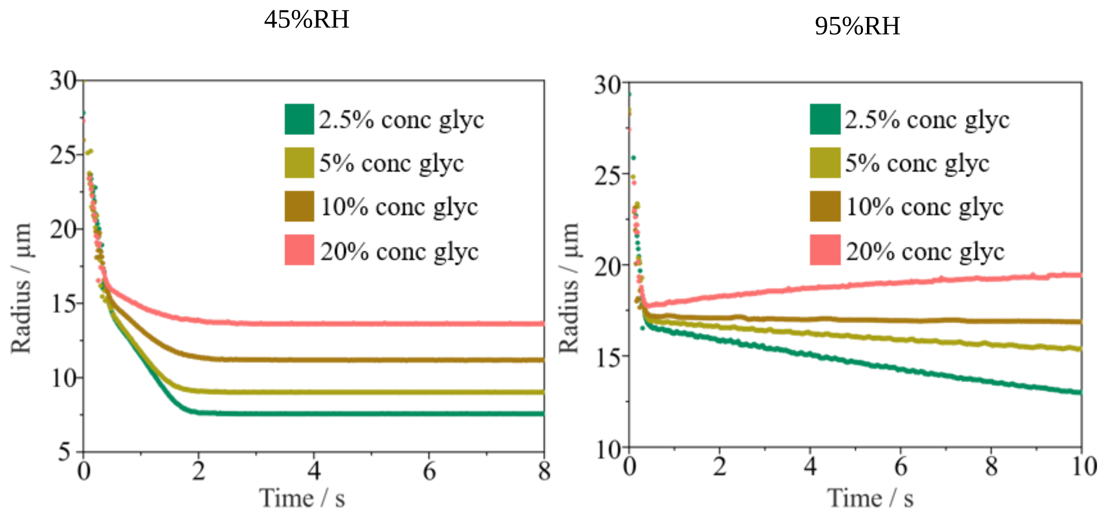

Figure S2. Ethanol and glycerol droplets of varying concentration trapped in ambient and wet conditions. At 95%RH and 20% conc glycerol the droplet grows after the initial phase of water condensation due to the evaporative cooling. All other droplets reach a final radius below the inflexion point radius.

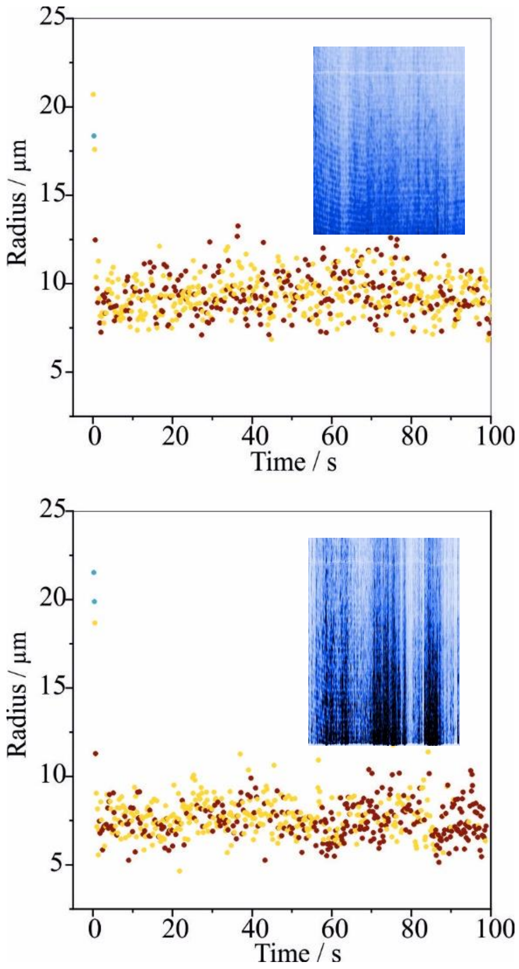

Figure S3. Top: glycerol0% solution at >100%RH on the specialised EDB . Bottom: glycerol50% solution at >100% on the specialised EDB . No dissolution seen for either system.
